# Self-assembly of gelatin and collagen in the polyvinyl alcohol substrate and its influence on cell adhesion, proliferation, shape, spreading and differentiation

**DOI:** 10.3389/fbioe.2023.1193849

**Published:** 2023-07-14

**Authors:** I-Chi Wu, Je-Wen Liou, Chin-Hao Yang, Jia-Hui Chen, Kuan-Yu Chen, Chih-Huang Hung

**Affiliations:** ^1^ Institute of Medical Sciences, Tzu Chi University, Hualien City, Taiwan; ^2^ Plastic Surgery Division, Surgical Department, Hualien Armed Forces General Hospital, Hualien City, Taiwan; ^3^ Department of Biochemistry, School of Medicine, Tzu Chi University, Hualien City, Taiwan; ^4^ Department of Surgery, Taipei Tzu Chi Hospital, New Taipei City, Taiwan; ^5^ Department of Surgery, New Taipei City Hospital, New Taipei City, Taiwan

**Keywords:** polyvinyl alcohol, collagen, gelatin, stem cells, adhesion

## Abstract

Culture substrates display profound influence on biological and developmental characteristic of cells cultured *in vitro*. This study investigates the influence of polyvinyl alcohol (PVA) substrates blended with different concentration of collagen or/and gelatin on the cell adhesion, proliferation, shape, spreading, and differentiation of stem cells. The collagen/gelatin blended PVA substrates were prepared by air drying. During drying, blended collagen or/and gelatin can self-assemble into macro-scale nucleated particles or branched fibrils in the PVA substrates that can be observed under the optical microscope. These collagen/gelatin blended substrates revealed different surface topography, z-average, roughness, surface adhesion and Young’s modulus as examined by the atomic force microscope (AFM). The results of Fourier transform infrared spectroscopy (FTIR) analysis indicated that the absorption of amide I (1,600–1,700 cm^−1^) and amide II (1,500–1,600 cm^−1^) groups increased with increasing collagen and gelatin concentration blended and the potential of fibril formation. These collagen or/and gelatin blended PVA substrates showed enhanced NIH-3T3 fibroblast adhesion as comparing with the pure PVA, control tissue culture polystyrene, conventional collagen-coated and gelatin-coated wells. These highly adhesive PVA substrates also exhibit inhibited cell spreading and proliferation. It is also found that the shape of NIH-3T3 fibroblasts can be switched between oval, spindle and flattened shapes depending on the concentration of collagen or/and gelatin blended. For inductive differentiation of stem cells, it is found that number and ration of neural differentiation of rat cerebral cortical neural stem cells increase with the decreasing collagen concentration in the collagen-blended PVA substrates. Moreover, the PVA substrates blended with collagen or collagen and gelatin can efficiently support and conduct human pluripotent stem cells to differentiate into Oil-Red-O- and UCP-1-positive brown-adipocyte-like cells via ectodermal lineage without the addition of mitogenic factors. These results provide a useful and alternative platform for controlling cell behavior *in vitro* and may be helpful for future application in the field of regenerative medicine and tissue engineering.

## Introduction

Culture substrates made from naturally-derived or synthetic materials can display profound influences on the biological or developmental characteristics of cells *in vitro* (Lutolf and Hubbell., 2005; Alves et al., 2010). Hence, engineering an appropriate culture substrate to direct cellular behavior and function is a very important issue in the field of regenerative medicine and tissue engineering (Langer and Tirrell, 2004; Kohane and Langer, 2008).

Among the many synthetic materials that can be used to prepare culture substrates, polyvinyl alcohol (PVA) has been reported to be bioinertness (Naahidi et al., 2017) and is one of the widely utilized materials in the field of biomedical application due to its high water content and soft tissue-like elasticity (Chiellini et al., 2003; Baker et al., 2012). However, owing to the lack of cell adhesion on their surface and intrinsic highly hydrophilic properties, PVA has been shown to resist protein adsorption and cell adhesion (Baker et al., 2012; Ino et al., 2013), and as a result, cultured cells are unable to anchor and survive on the PVA substrates (Nuttelman et al., 2001; Schmedlen et al., 2002). To overcome the anchoring problem, PVA are often grafted or blended with adhesion-enhancing nature polymers for obtaining novel biomaterials with improved biocompatibility. Chuang et al. (1999) demonstrated that PVA/chitosan blended substrates increase cell adhesion and growth and found that fibroblasts cultured on the PVA/chitosan blended substrates had good spreading, cytoplasm webbing and flattening and were more compacting than on the pure PVA substrate. Other studies also demonstrated that PVA blended with collagen, hyaluronic acid, hydroxyapatite or alginate can sustain chondrocyte adhesion and proliferation with enhanced mechanical performance (Oh et al., 2016; Chocholata et al., 2020; Lan et al., 2020). Gelatin is also one of the natural polymers that has been used for blending to alter material structure, enhance material biocompatibility as well as facilitate cell adhesion, migration, proliferation and differentiation (Zulkiflee and Fauzi, 2021). Zhang et al. (2022) showed that PVA/gelatin blended substrates, prepared via dry-annealing method, increased protein adsorbing capability, cell adhesion area and ratio of non-spherical cells with the increment of gelatin concentration. Ino et al. (2013) also demonstrated that PVA/gelatin blended substrate can be formed via chemically crosslinking and the cell adhesion of endothelial cells was obviously different depending on the ratios of PVA and gelatin.

The plasticity and self-renewing properties of stem cells make them a valuable tool in the field of regenerative medicine. The fate of stem cells can be regulated by both biochemical and physical factors (Xing et al., 2019). Biochemical factors such as soluble growth factors and insoluble extracellular matrix are known to affect the differentiation of stem cells. Physical factors such as substrate stiffness, topography, alignment and mechanical force can also influence stem cell differentiation. Several studies revealed that stiffness plays an important role for conducting differentiation of stem cells (Lv et al., 2015). Evans et al. (2009) demonstrated that by growing embryonic stem cells on flexible polydimethylsiloxane substrates with varying stiffness, osteogenic differentiation of embryonic stem cells was enhanced on stiff substrates compared to soft substrates. Engler et al. (2006) also demonstrated human mesenchymal stem cells cultured on soft substrates (0.1–1 kPa) that mimic brain tissue are neurogenic, stiffer substrates (8–17 kPa) that mimic muscle tissue are myogenic, and comparatively rigid substrates (25–40 kPa) that mimic collagenous bone prove osteogenic. Saha et al. (2008) developed a synthetic and interfacial hydrogel culture system to assess the effects of substrate moduli on the behavior of adult neural stem cells and demonstrated that in serum-free neuronal differentiation media, a peak level of the neuronal marker β-tubulin III, was observed on substrate modulus of 500 Pa, near the physiological stiffness of brain tissue. This study also found that under mixed neuronal and glial differentiation conditions with serum, softer substrates (∼100–500 Pa) promote neural differentiation, whereas harder substrates (∼1,000–10,000 Pa) favored glial differentiation. Oh et al. (2016) demonstrated that, from an *in vitro* cell culture using the stiffness gradient PVA/HA substrates, it was observed that human bone marrow mesenchymal stem cells have favorable stiffness ranges for induction of differentiation into specific cell types (∼20 kPa for nerve cells, ∼40 kPa for muscle cells, ∼80 kPa for chondrocytes, and ∼190 kPa for osteoblasts). These results suggest that substrate stiffness can mimic physiological stiffness to direct stem cell differentiation towards the corresponding cell types.

Although many substrates have been created for studying cell adhesion, most of these methods involve complicated experimental procedures or expensive materials and equipment (Padavan et al., 2011; Millon et al., 2012; Rafat et al., 2012). Hence, it is still highly desirable to have a simply prepared and low-cost substrate preparation. In this study, substrates are prepared using PVA blended with adhesion-enhancing nature polymers collagen or/and gelatin by a relative simple method, air drying. The first aim of this study is to examine whether PVA/collagen/gelatin blended substrates can improve cell adhesion of pure PVA and also alter their topographical and mechanical properties. Because PVA-based induction of stem cell differentiation is few documented and since PVA are common known as soft tissue replacement (Duque-Ossa et al., 2021), the second aim of this study is to investigate whether soft PVA substrates have the potential to direct stem cell differentiation towards cells of soft tissue.

## Materials and methods

### Preparation of the PVA, PVA-G, PVA-C, and PVA-CG polymer substrates

Commercially available PVA (BP-17 and BF-17, Chang Chun, Taiwan) was dissolved in H_2_O at 90°C to form 3.5% and 7% (wt/wt) PVA polymer solutions. After cooling, the 7% PVA polymer solution was then blended with 40 mg/mL, 20 mg/mL, 10 mg/mL, 5 mg/mL, 2 mg/mL, and 1 mg/mL gelatin solution (dissolved in H_2_O) in the 1:1 volume ratio to give rise to different gelatin concentrations of the PVA-G polymer solution [PVA-G (20 mg/mL), PVA-G (10 mg/mL), PVA-G (5 mg/mL), PVA-G (2.5 mg/mL), PVA-G (1 mg/mL), and PVA-G (0.5 mg/mL)]. The 3.5% PVA polymer solution was blended with collagen to give rise to different collagen concentrations of the PVA-C polymer solution [PVA-C (200 ug/mL), PVA-C (100 ug/mL), PVA-C (50 ug/mL), and PVA-C (25 ug/mL)]. The PVA-CG polymer solution was prepared by adding collagen to the different concentration of the PVA-G polymer solution to give rise to different gelatin and collagen concentration of the PVA-CG polymer solution. For example, PVA-C (100 ug/mL)-G (5 mg/mL) denotes that the PVA-CG polymer solution has the final concentration of 3.5% PVA, 100 ug/mL collagen and 5 mg/mL gelatin dissolved in water. For cell cultures, 0.1 mL PVA, PVA-G, PVA-C, and PVA-CG polymer solution were coated in each 24-culture-well (area: 1.9 cm^2^) and dried for 7 days at room temperature as thin films.

### Cell culture

To test the biocompatibility (adhesion and viability) of prepared PVA, PVA-G, PVA-C, and PVA-CG polymer substrates, NIH-3T3 cell line was used. This cell line was cultured in the DMEM/High Glucose medium containing 10% fetal bovine serum (FBS), penicillin G (100 IU/mL), and streptomycin (100 μg/mL) at the cell density of 1.5 × 104/cm^2^. Cultures were maintained at 37°C in a humidified atmosphere of 95% air/5% CO_2_ and were subpassaged every 3–4 days.

### Maintenance and differentiation of human embryonic stem cells

Human embryonic stem cell line (TW1), which was gifted from Dr. Dah-Ching Ding of Tzu Chi University and can be obtained from Bioresource Collection and Research Center (BCRC), Taiwan (Cheng et al., 2008; Ding et al., 2012; Chang et al., 2017), was maintained on mitomycin-treated mouse embryonic fibroblast (MEF) feeder layers in the embryonic stem cell culture medium [DMEM/F12 supplemented with 20% (vol/vol) KnockOut serum replacement (Invitrogen), 1 mM nonessential amino acids (Invitrogen), Glutamax (Invitrogen), penicillin/streptomycin (Invitrogen), 0.55 mM 2-mercaptoethanol (Invitrogen), 4 ng/mL recombinant human FGF2 (R&D Systems)] (Thomson et al., 1998; Hoffman and Carpenter, 2005). Cultures were manually passaged at a 1:6–1:8 split ratio every 5–7 days.

MEF were harvested from E13-E14 embryos of female ICR mice and cultured in Dulbecco’s modified Eagle’s medium (DMEM; Invitrogen) supplemented with 5% fetal bovine serum (FBS; Gibco), 2 mmol/l l-glutamine (Invitrogen) and penicillin/streptomycin (Invitrogen). For use as feeder cells, MEF were inactivated with 10 μg/mL mitomycin C (Sigma) for 3 h to arrest mitosis, thoroughly washed in phosphate buffered saline (PBS), and replated at 3 × 104 cells/cm^2^ on 0.1% gelatin-coated tissue culture plates and reached confluence in 3–4 days (Cheng et al., 2008).

Human embryonic stem (ES) cells were then cultured on the PVA, PVA-C, PVA-G, and PVA-CG polymer substrates in the ES medium to observe if these blended PVA substrates could serve as mouse embryonic fibroblast feeder layer to maintain the self-renewing of human ES cells. For differentiation, the embryonic stem cell culture medium was replaced with FBS-containing medium (DMEM/F12 containing 10% FBS, penicillin/streptomycin) to see whether human ES cells can attach, grow and be induced to differentiate on the blended PVA substrates prepared in this study.

### Measurement of cell adhesion and viability

The cell adhesion and viability are measured by the method of the MTT assay to assess the ability of attachment, proliferation and survival of NIH-3T3 and TW1 cells on the PVA, PVA-G, PVA-C, and PVA-CG polymer substrates. The seeding density of NIH-3T3 for MTT test is 1.5 × 104/cm^2^. At indicated time points (4 h, 1 day, 2 days, and 4 days), the culture medium was removed and incubated with 0.1 mL of MTT solution (2 mg/mL) for 3 h at 37°C. After incubation, the MTT solution was aspirated and the formazan reaction products were dissolved in dimethyl sulfoxide and shaken for 30 min. The optical density of the formazan solution was read on an ELISA plate reader at 570 nm.

### Atomic force microscope

AFM experiments were performed with JPK AFM Nano-wizard (Bruker Nano, Berlin, Germany). The AFM probe used was Aluminium reflex coating of cantilevers with silicon rotated tips (ContAI, Budgetsensors, Bulgaria). The spring constant for the cantilevers was 0.2 N/m, half angle 20 deg. and a tip radius of 10 nm. The AFM images were captured in contact mode at room temperature in dry environment and the resolution is 512 × 512 pixels. The scanning rate was set to 1 Hz. Both the height image and deflection image were recorded. Average Roughness values (Ra) and Z-average were then measured using JPKSPM data processing software. The measurement size was 100 um × 100 um. Before the force curves acquisition, the precise spring constant of the cantilever was calibrated by the thermal noise method in the air. To measure Young’s modulus and adhesion force, the force curves were recorded within a scan area of 100 um × 100 um, within which a grid range was 8 × 8 points. In total, 64 force curves were recorded. The indentation rate was set to 2 um/s. The batch processing of the curves was using baseline subtraction, contact point correction, vertical tip position correction, and applying elasticity fit. The Young’s modulus values were obtained by fitting Hertz/Sneddon model for cone indenter with half cone angle 20 deg. (Akbarzadeh Solbu et al., 2022; Asgari et al., 2022).

### FTIR analysis

FTIR-ATR (Nicolet iS5, Thermo Fisher Scientific, United States) was used to confirm the absorption bands of amide I (1,600–1,700 cm^−1^) and amide II (1,500–1,600 cm^−1^) groups, the most prominent absorption bands of proteins, of the prepared PVA substrates. Briefly, samples are dried in a desiccator at R.T. and were sandwiched between the reflection crystal (KRS-5) and the back plate. Spectra were taken with air as the background using OMNIC spectra software (OMNIC 9.12.1019, Thermo Fisher Scientific, United States) in the range of 400–4,000 cm^−1^ (Schwaighofer and Lendl, 2023).

### RT-PCR

After the human ES cells were seeded on the control TCPS wells or blended PVA polymer substrates, total cellular RNA was extracted at indicated time points using Trizol reagent (REzol, Protech, Taipei, Taiwan) according to the manufacturer’s instructions. After cells were lysed, they were transferred to centrifuge tubes. Chloroform (0.2 mL) was then added to each tube and mixed with the sample. The sample tubes were incubated at room temperature for 5 min, and centrifuged at 12,000 *g* for 15 min at 4°C. The aqueous phase was transferred to new tubes, each containing 500 μL isopropanol. Samples were centrifuged at 12,000 *g* for 10 min at 4°C. The supernatant was removed, and the RNA pellet was washed with 500 μL 75% ethanol. The RNA pellet was then dissolved in 20 μL water containing 0.01% DEPC and stored in a −80°C freezer for further use. cDNA was prepared from RNA by using a reverse-transcription kit (Bio-Rad, Hercules, CA, United States). All PCR samples were analyzed by electrophoresis on 2% agarose gel containing 0.5 μg/mL ethidium bromide (Sigma). The primers used in this study are: β-actin (5′-CAC​CTT​CTA​CAA​TGA​GCT​GCG-3′, 5′-TGC​TTG​CTG​ATC​CAC​ATC​TGC-3′), Adpsin (5′-GCG​CAC​CTG​GCG​GCG​TCC​TG-3′, 5′-GCA​CTG​CGC​GCA​GCA​CGT​CGT​A-3), Gata4 (5′-CTA​CAG​GGG​CAC​TTA​ACC​CA-3′, 5′-AGA​GCT​GAA​TCG​CTC​AGA​GC-3), Gata6 (5′-CCT​CAC​TCC​ACT​CGT​GTC​TGC-3′, 5′-GTC​CTG​GCT​TCT​GGA​AGT​GG-3), GLUT4 (5′-GCC​ATT​GTT​ATC​GGC​ATT​CT-3′, 5′-CTA​CCC​CTG​CTG​TCT​CGA​AG-3′), hALBP (5′-AGT​CAA​GAG​CAC​CAT​AAC​CTT​AGA-3′, 5′-CCT​TGG​CTT​ATG​CTC​TCT​CAT​AA-3′), Sox9 (5′-GCC​ACG​GAG​CAG​ACG​CAC-3′, 5′-GCG​CCT​GCT​GCT​TGG​ACA-3′), Sox10 (5′-GAC​CGC​CGC​CAT​CCA​GGC-3′, 5′-AGT​AGC​TGC​TCA​CAT​GGC​CT-3′).

### Oil red O staining

At indicated time points, the cultured cells were rinsed with phosphate buffered saline (PBS) three times, fixed with 4% paraformaldehyde for 30 min at 4°C, and then stained with filtered Oil Red O solution (0.5% Oil Red O in isopropyl alcohol) for 2 h at room temperature. After staining, the cells were washed three times with PBS and observed under an optical microscope. To quantify the percentage of Oil-Red-O-positive adipocytes, human ES cells differentiated on the PVA-CG polymer substrates were costained with Oil Red O and DAPI.

### Indirect immunofluorescence

At indicated time points, cultured cells were fixed in ice-cold 4% paraformaldehyde in PBS for 20 min and washed three times in PBS. After fixing, cells were incubated with primary antibodies diluted in PBS containing 0.5% triton-X-100% and 10% bovine serum albumin for 24 h at 4°C. The primary antibodies and their dilution used in this study were mouse anti-microtubule-associated protein 2 polyclonal antibody (anti-MAP2; 1:500; Millipore), rabbit anti-glial fibrillary acidic protein polyclonal antibody (anti-GFAP; 1:500; Chemicon), goat anti-uncoupling protein 1 polyclonal antibody (anti-UCP1; 1:1,000; Santa Cruz), rabbit anti-nestin polyclonal antibody (anti-nestin; 1:500; Chemicon), rabbit anti-brachyury T polyclonal antibody (1:800, Abcam), mouse anti-α-fetoprotein monoclonal antibody (1:500; Millipore), rabbit anti-forkhead box D3 polyclonal antibody (anti-FoxD3; 1:1,000, Aviva), mouse anti-stage-specific embryonic antigen-4 (anti-SSEA4; 1:500, Chemicon), rabbit anti-octamer 4 polyclonal antibody (anti-OCT4, 1:500, Chemicon), mouse anti-cytokeratin 15 monoclonal antibody (anti-K15, 1:500, Invitrogen). FITC- and rhodamine-conjugated secondary antibodies were used to visualize the signal by reacting with cells for 1 h at room temperature. Cell morphology was viewed with a photomicroscope (Zeiss LAMBDA 10–2, Germany). Cells were also counterstained with DAPI. Immunostained cells were visualized by indirect immunofluorescence under the fluorescent microscope (Axiovert 100TV, Germany).

### Measurement of particle size, fibril length, fibril covering area and cell spreading area

Digital photomicrographs were taken in random fields at indicated experimental points and conditions. Quantification of collagen particle size was evaluated by the diameter of the particles. Total 30–50 independent readings of the particle diameter, fibril length, fibril covering area and cell spreading area were measured using NIH Image software (ImageJ), and the means and standard deviation were also calculated.

### Isolation and culture of cortical neural stem cells

Cortical neural stem cells were prepared from pregnant Wistar rat embryos on day 14–15 according to a protocol detailed previously (Tsai and McKay, 2000). Briefly, rat embryonic cerebral cortices were dissected, cut into small pieces and mechanically triturated in cold Hank’s balanced salt solution (HBSS) containing 0.4 g/L KCl, 0.09 g/L Na_2_HPO_4_, 7H_2_O, 0.06 g/L KH_2_PO_4_, 0.35 g/L NaHCO_3_, 0.14 g/L CaCl_2_, 0.10 g/L MgCl_2_.6H_2_O, 0.10 g/L MgSO_4_.7H_2_O, 8.0 g/L NaCl, and 1.0 g/L D-glucose in deionized water. The dissociated cells were collected by centrifugation and were resuspended in a serum-free medium (SFM) containing DMEM-F12, 8 mM glucose, glutamine, 20 mM sodium bicarbonate, 15 mM HEPES and N2 supplement (25 mg/mL insulin, 100 mg/mL human apotransferri, 20 nM progesterone, 30 nM sodium selenite, pH 7.2). The number of live cells was counted by trypan blue exclusion assay in a hemocytometer. Cerebral cortical neural stem cells were purified and cultured in T25 culture flasks (Corning, New York, United States) at a density of 50,000 cells/cm^2^ in the above culture medium in the presence of bFGF at a concentration of 20 ng/mL. Cultures were maintained at 37°C in a humidified atmosphere of 95% air/5% CO_2_. After 1–3 days of culture *in vitro*, cells underwent division and formed clust ers of cells, termed neurospheres, which suspended in the medium. Subsequently, adherent cells were discarded and suspended neurospheres were collected by centrifugation, mechanically dissociated and subcultured as single cell in a new T25 culture flask at a density of 50,000 cells/cm^2^ in the fresh culture medium containing the same concentration of bFGF. These cells grew into new spheres in the subsequent 2–3 days. The procedure of subculture was repeated 2–3 weeks to achieve the purified cortical neural stem cells and proliferating neurospheres. The plasticity of these purified cortical neural stem cells was identified by the method of immunocytochemistry with anti-nestin, anti-NSE, and anti-GFAP, which has been reported in our previous publication^22^.

### Western blot analysis

The protein expression of MAP-2, GFAP, and β-actin was determined at day 7 of cell culture. Rat cerebral cortical neurospheres cultured on the substrates were resuspended in cell lysis buffer (Thermo scientific) and sonicated. After centrifugation, the protein content was determined in the supernatants by a BCA protein quantification kit (Pierce Biotechnology, Rockford, IL). Sixty mg proteins from neurospheres were added to Laemmli sample buffer and boiled for 10 min. Proteins were separated by sodium dodecyl sulfate-polyacrylamide gel electrophoresis (SDS-PAGE) and blotted onto polyvinylidene difluoride membranes. Western blotting was performed using anti-MAP-2 (Chemicon), anti-GFAP (Chemicon), and anti-β-actin (Chemicon). The membranes were incubated with the primary antibodies overnight at 4°C. After extensive washing, the membranes were further incubated with horseradish peroxidase-conjugated secondary antibodies for 1 h. The blottings were then developed using an enhanced chemiluminescence detection system (Millipore).

### Statistical analysis

All measurements were presented as means ± S.D. (standard deviation). Statistical significance was calculated using one-way analysis of variance (ANOVA) or using independent-samples Student’s *t*-test. The *p* values of <0.05 were considered statistically significant.

## Results

### Different surface structure of the PVA, PVA-C, PVA-G, and PVA-CG substrates

**FIGURE 1:**
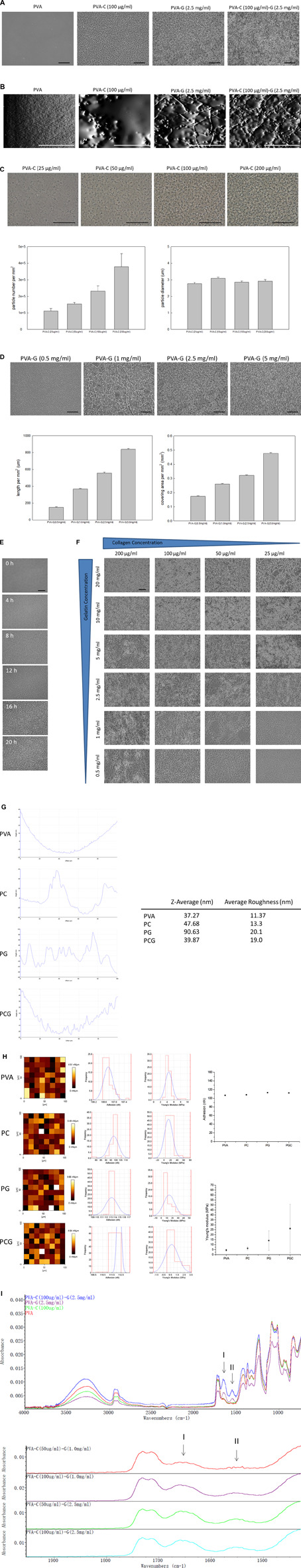
(Continued) The topography, z-average, average roughness, surface adhesion force, Young’s modulus and FTIR analysis of the PVA, PVA-C, PVA-G, and PVA-CG substrates. **(A,B)** The topography of the PVA, PVA-C (100 μg/mL), PVA-G (2.5 mg/mL), and PVA-C (100 μg/mL)-G (2.5 mg/mL) substrates were examined under the **(A)** optic microscope and **(B)** atomic force microscope. **(C)** The morphology, number, and diameter of nucleated particles of the PVA-C substrates blended with different concentration of collagen (25, 50, 100, and 200 μg/mL). **(D)** The morphology, length, and covering area of elongated fibrils of the PVA-G substrates blended with different concentration of gelatin (0.5, 1.0, 2.5, and 5.0 mg/mL). **(E)** Time-series observation of the surface topography of the PVA-C (100 μg/mL)-G (2.5 mg/mL) substrates after 0 h, 4 h, 8 h, 12 h, 16 h, and 20 h air drying. **(F)** The surface topography of the PVA-CG substrates blended with different concentration of collagen (25, 50, 100, and 200 μg/mL) and gelatin (0.5, 1.0, 2.5, 5.0, 10.0, and 20.0 mg/mL). **(G)** The z-average and average roughness of the PVA, PVA-C (100 μg/mL), PVA-G (2.5 mg/mL), and PVA-C (100 μg/mL)-G (2.5 mg/mL) substrates. **(H)** The surface adhesion force and Young’s modulus of the PVA, PVA-C (100 μg/mL), PVA-G (2.5 mg/mL), and PVA-C (100 μg/mL)-G (2.5 mg/mL) substrates. **(I)** Upper: FTIR analysis of the PVA, PVA-C (100 μg/mL), PVA-G (2.5 mg/mL), and PVA-C (100 μg/mL)-G (2.5 mg/mL) substrates. Bottom: FTIR analysis of the PVA-C (50 μg/mL)-G (1.0 mg/mL), PVA-C (100 μg/mL)-G (1.0 mg/mL), PVA-C (50 μg/mL)-G (2.5 mg/mL), and PVA-C (100 μg/mL)-G (2.5 mg/mL) substrates. Scale bar = 50 μm.

Previous studies have demonstrated that, owing to the intrinsic properties of the PVA substrates, several anchoring-dependent cells cannot attach and survive upon the PVA substrates *in vitro* (Nuttelman et al., 2001; Schmedlen et al., 2002; Baker et al., 2012). Hence, in order to modify these effect of PVA substrates, we blended PVA substrates with extracellular matrixes collagen or/and gelatin to enhance the cell anchoring and survival on the PVA substrates. After blending, coating and drying, it is observed that these non-blended and blended PVA substrates show different surface topography (Figure 1). Without blending collagen and gelatin, PVA substrates reveal the dense and smooth surface structure similar with previous published results (Ino et al., 2013) (Figure 1A). Interestingly, it is found that after blending, collagen or/and gelatin can self-assemble to form nucleated particles or branched fibrils in the PVA substrates with the scale that can be observed under the optical microscope (Figure 1A). While the surface of PVA substrates blended with collagen (PVA-C) display nucleated particles, the surface of PVA substrates blended with gelatin (PVA-G) show the branched and elongated fibril structure (Figure 1A). Additionally, the surface of PVA substrates blended with collagen and gelatin (PVA-CG) show branched and intermingled fibrils (Figure 1A). These nucleated particles and branched fibrils observed on the PVA-C, PVA-G, and PVA CG substrates cannot be found on the collagen-coated or gelatin-coated wells (data not shown). The micrographs examined under the atomic force microscope also revealed that these nucleated particles and branched fibrils are embedded within the PVA substrates (Figure 1B). In PVA-C substrates, the number of nucleated collagen particles increases with the amount of collagen blended and are significantly different between groups (*p* < 0.05); however, the size of these particles does not change significantly among these groups (Figure 1C). In PVA-G substrates, when blended with the lower gelatin concentration (0.5 mg/mL), nucleated gelatin particles are formed in the PVA substrates (Figure 1D). However, as the blended gelatin concentration increased, these nucleated gelatin particles start to assemble or connect together to form branched and elongated fibrils; quantitatively, the total length and covering area per mm^2^ of elongated gelatin fibrils in the PVA substrates increase with the amount of gelatin blended and are significantly different between groups (*p* < 0.05) (Figure 1D). Time-series observation shows that the number of branched and elongated fibrils in the PVA-CG substrates increases with time during the substrate preparation (Figure 1E), and the amount of branched fibrils in the PVA-CG substrates increases with the amount of collagen and gelatin blended (Figure 1F). According to the AFM analysis, it is found that the z-average and average roughness increased along with the formation of nucleated particles and branched fibrils as comparing to the non-modified PVA substrates. The z-average of PVA, PVA-C, PVA-G, and PVA-CG substrates is 37.27, 47.68, 90.63, and 39.87 nm, respectively (Figure 1G). The average roughness of PVA, PVA-C, PVA-G, and PVA-CG substrates is 11.37, 13.3, 20.1, and 19.0 nm, respectively (Figure 1G). It is also found that surface adhesion force showed no difference between PVA, PVA-C, PVA-G, and PVA-CG groups (Figure 1H). However, the Young’s modulus increased along with the particle and fibril formation in the PVA-C, PVA-G, and PVA-CG groups (Figure 1H). The spectra of FTIR analysis showed that increased band absorbance of the amide I band (1,600–1,700 cm^−1^-originating from the C=O stretching and N-H in-phase bending vibration of the amide group) and the amide II band (1,500–1,600 cm^−1^-arising from N-H bending and C-N stretching vibrations) in PVA-G and PVA-CG groups but not in the PVA-C group (Figure 1I; upper). Furthermore, It is found that the absorbance of amide I and amide II bands increased with the increasing collagen and gelatin concentration blended in the PVA substrates (Figure 1I; bottom). These results suggested that the presence and intercalation of collagen or/and gelatin in the PVA substrates after blending, and the assembly of collagen or/and gelatin into particles or fibrils may influence substrates’ surface topographical and mechanical properties.

### Improved cell adhesion and viability of NIH-3T3 cells on the PVA-C, PVA-G, and PVA-CG substrates

**FIGURE 2:**
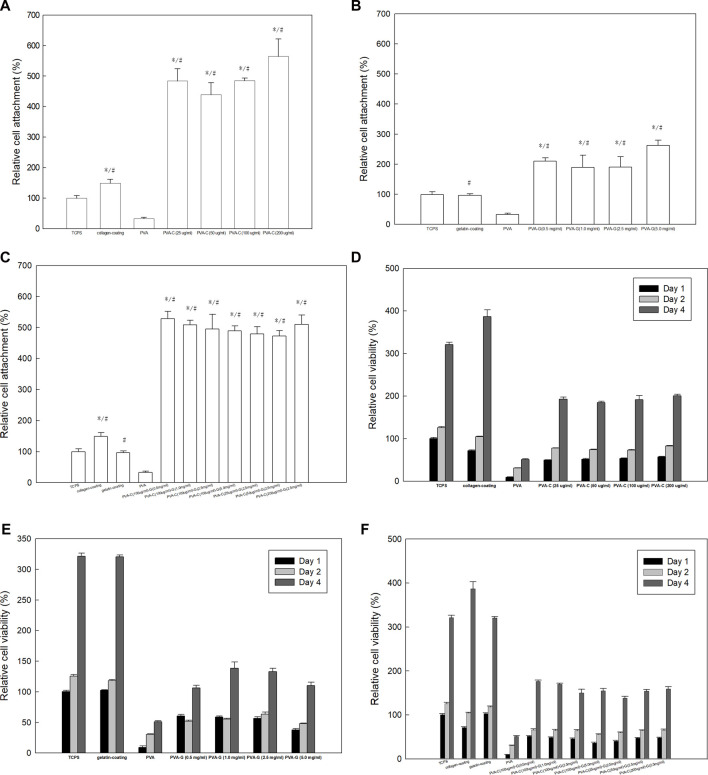
Improved adhesion and viability of NIH-3T3 cells on the PVA-C, PVA-G, and PVA-CG substrates. MTT assay of NIH-3T3 cells on **(A,D)** PVA-C, **(B,E)** PVA-G, and **(C,F)** PVA-CG substrates after **(A,B,C)** 4 h, **(D,E,F)** 1 day, 2 days, and 4 days of culture as comparing with PVA substrates, control TCPS, conventional collagen-coating and gelatin-coating wells. Graph represents the mean MTT absorbance (O.D. 570–O.D. 630; normalized to the absorbance of the control TCPS wells at 4 h) ± s.e.m. from 6 independent determinations. * and # respectively denotes significant difference (*p* < 0.05) of the MTT reduction activity compared to that on the control TCPS wells and PVA substrates as determined by Student’s *t*-test.

In order to investigate if these modified PVA-C, PVA-G, and PVA-CG substrates can improve anchoring and survival of cultured cells *in vitro*, we cultured NIH-3T3 fibroblasts on these modified substrates and examine the attachment and viability of cells by means of the MTT colorimetric assay. Quantitatively, as indicated by the results of MTT assay after 4 h of culture, the cell attachment were improved on the PVA-C, PVA-G, and PVA-CG substrates as comparing with those on the TCPS, non-blended PVA, collagen-coated and gelatin-coated wells (Figures 2A–C). Furthermore, the results of MTT assay after 1 day, 2 days, and 4 days of the culture show that the viability of NIH-3T3 cells on the PVA-C, PVA-G, and PVA-CG substrates increased with time, which suggested that these modified PVA substrates are biocompatible for NIH-3T3 cells *in vitro* (Figures 2D–F). Although the viability of NIH-3T3 cells on the PVA-C, PVA-G, and PVA-CG substrates are improved and significantly higher than that on the non-blended PVA substrates, they are not higher than those on the control TCPS, gelatin-coated or collagen-coated wells during the 4-day culture *in vitro*, assuming that NIH-3T3 cells are more adhesive but less proliferated on the modified PVA-C, PVA-G, and PVA-CG substrates.

### Controllable cell shape and spreading of NIH-3T3 cells on the PVA-C, PVA-G, and PVA-CG substrates

**FIGURE 3:**
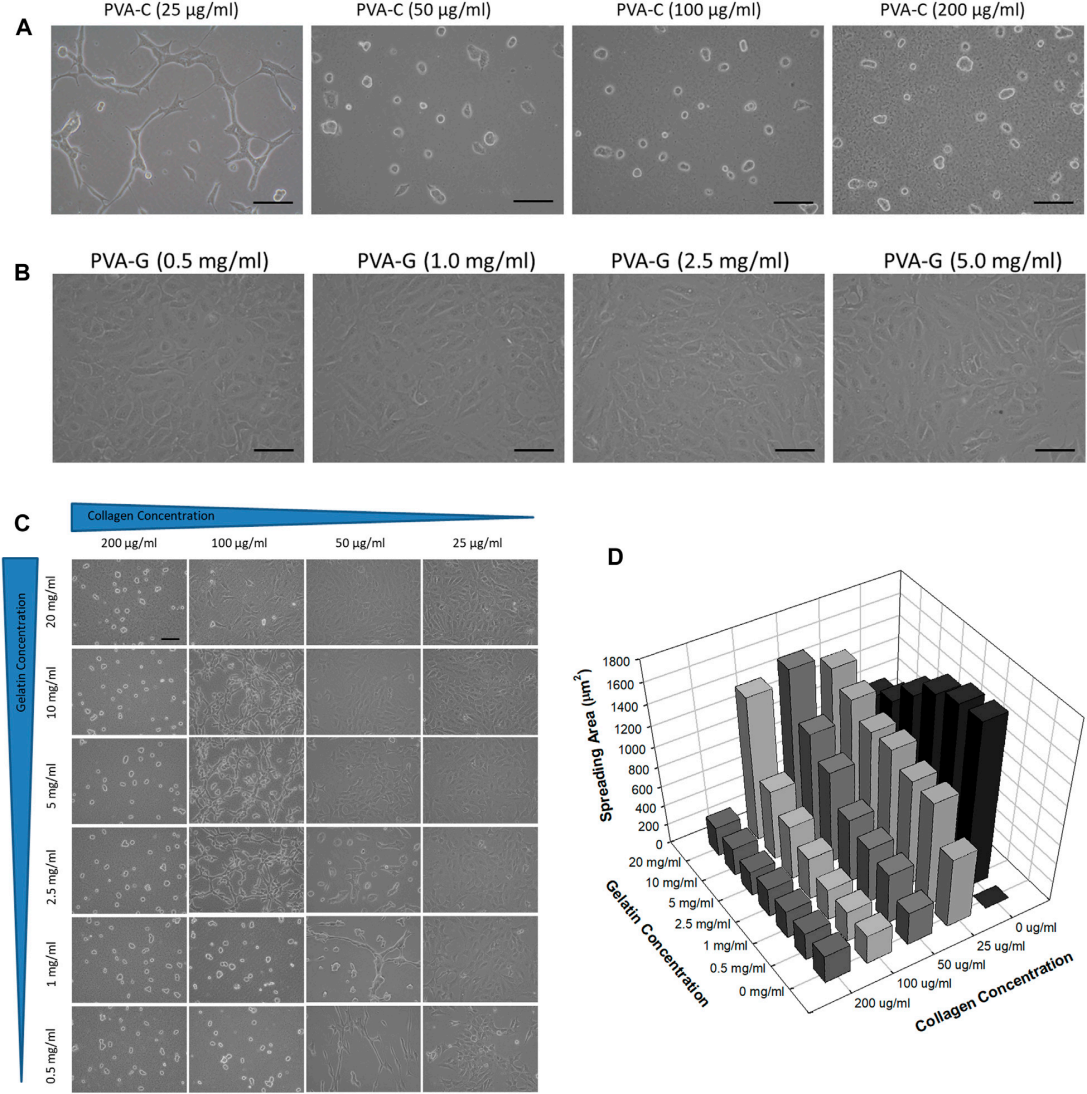
Controlled cell shape and spreading of NIH-3T3 cells on the PVA-C, PVA-G, and PVA-CG substrates. Cell morphology of NIH-3T3 cells on the **(A)** PVA-C, **(B)** PVA-G, and **(C)** PVA-CG substrates blended with different concentration of collagen and gelatin. Scale bar = 25 μm. **(D)** Quantification of spreading area of NIH-3T3 cells on the PVA-CG substrates blended with different concentration of collagen and gelatin.

As predicted, NIH-3T3 cells cannot attach and survive on the non-blended PVA substrates after 4 days of culture *in vitro* (data not shown). However, it is found that these cells can now adhere onto the PVA-C substrates and their morphological appearances are dependent on the concentration of collagen blended. Generally, NIH-3T3 cells cultured on the PVA-C polymer substrates blended with higher concentration of collagen (200, 100, and 50 μg/mL) reveal round and oval morphology without any cytoplasmic extension (Figure 3A). Instead, NIH-3T3 cells cultured on the PVA-C substrates blended with lower concentration of collagen (25 μg/mL) exhibit spindle and chained morphology (Figure 3A). On the contrary, the morphological appearances of attached NIH-3T3 cells on the blended PVA-G substrates show flatten spreading morphology exclusively regardless of the concentration of gelatin blended (Figure 3B). Likewise, NIH-3T3 cells also can attach on the PVA-CG substrates. Interestingly, three different types of cell morphology can be observed on the PVA-CG substrates (Figure 3C). In general, NIH-3T3 cells exhibit flatten spreading morphology on the PVA-CG substrates when the relative amount of gelatin is dominant in the PVA-CG substrates. On the contrary, when the relative amount of collagen is dominant in the PVA-CG substrates, NIH-3T3 cells exhibit round and oval morphology. Additionally, NIH-3T3 cells reveal another intermediate spindle and chained morphology on the PVA-CG substrates when the relative amount of collagen and gelatin are adjusted and balanced in the indicated groups. It is also observed that the spreading area per cell of NIH-3T3 cells on the modified PVA-C, PVA-G, and PVA-CG substrates depends on the concentration of collagen or/and gelatin blended (Figure 3D). In the PVA-C and PVA-G substrates, the spreading area per cell increases with the decreasing collagen concentration or the decreasing gelatin concentration, respectively. In the PVA-CG substrates, the spreading area increases with the decreasing collagen concentration and increasing gelatin concentration. Taken together, these data suggested that PVA substrates blended with collagen or/and gelatin can be utilized as culture substrates to improve cell adhesion and viability as well as to control cell shape and spreading *in vitro*.

### Collagen-concentration dependent neuronal differentiation of rat cerebral cortical neural stem cells on the PVA-C substrates

**FIGURE 4:**
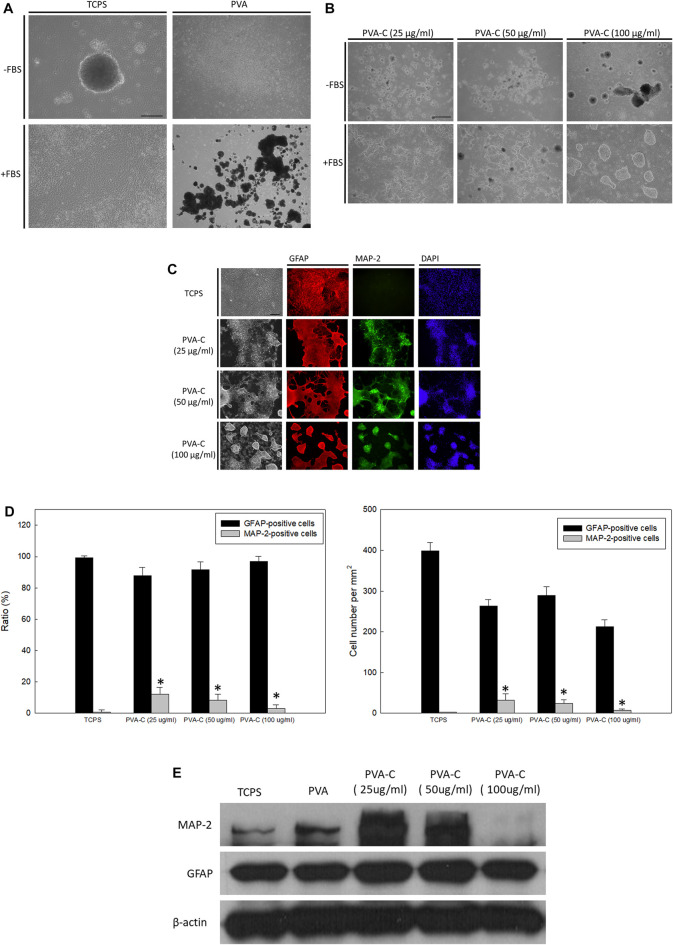
(Continued) Collagen-concentration dependent neuronal differentiation of rat cerebral cortical neural stem cells on the PVA-C substrates. **(A)** Rat cerebral cortical neurospheres cultured on the control tissue culture polystyrene (TCPS) wells and polyvinyl alcohol (PVA) substrates in the presence or absence of fetal bovine serum. **(B)** Rat cerebral cortical neurospheres cultured on the PVA-C substrates blended with different concentration of collagen (25, 50, and 100 μg/mL) in the presence or absence of fetal bovine serum. **(C)** Immunocytochemical stainings and **(D)** quantative analysis (ratio and absolute number) of neurosphere-derived MAP-2-positive neurons (green) and GFAP-positive glial cells (red) on the control TCPS wells and blended PVA-C substrates. DAPI (blue) represents the cell nuclear. * denotes significant difference (*p* < 0.05) compared to that on the control TCPS wells as determined by Student’s *t*-test. **(E)** Western blottings of MAP-2 and GFAP protein expression of neurospheres cultured on the control TCPS wells, PVA and blended PVA-C substrates. Scale bar = 50 μm.

For now, we have demonstrated that PVA substrates blended with collagen or/and gelatin have better cell attachment and viability than non-blended PVA substrates. In our previous study (Young and Hung, 2005; Hung and Young, 2006), it is found that rat embryonic cerebral cortical neural stem cells cannot attach, survive and differentiation on the non-blended PVA substrates. Hence, we next want to examine if these modified PVA substrates can exert influence on the *in vitro* behavior of cortical neural stem cells. Similar with our previous published studies, the cortical neural stem cells, cultured as neurospheres, can suspend and grow in the absence of fetal bovine serum (FBS) or be induced to attach and differentiate in the presence of FBS on the control TCPS wells (Figure 4A). However, regardless of the absence or presence of FBS, these neurospheres cannot attach and survive on the PVA substrates; instead, they suspended above the PVA substrates and are finally subjected to debris and death (Figure 4A). In this study, it is also observed that these neurospheres cannot attach and survive on the PVA-G and PVA-CG substrates regardless of the absence or presence of FBS, or the concentration of collagen or gelatin blended in the PVA substrates (data not shown). Nevertheless, the attachment of these neurospheres can be achieved on the PVA-C substrates in the presence of FBS (Figure 4B). Without the addition of FBS in the culture medium, these neurospheres also suspended on the PVA-C substrate regardless the concentrations of collagen blended in the PVA substrates (Figure 4B). Interestingly, the behavior of these FBS-required attached neurospheres on the PVA-C substrates is collagen-concentration dependent. That is, these neurospheres reveal unspreading morphology when they are cultured on the PVA-C substrates blended with higher concentration of collagen [PVA-C (100 μg/mL)] (Figure 4B). However, when the amount of collagen blended in the PVA-C substrates decreases [PVA-C (50 μg/mL) and PVA-C (25 μg/mL)], it can be observed that these neurospheres start to spread and a great number of cells migrate out from the neurospheres and differentiate into process-bearing cells on the modified PVA-C substrates (Figure 4B).

In order to visualize the neuronal or glial differentiation of rat cerebral cortical neural stem cells on the PVA-C substrates, attached cells are immunocytochemically stained with anti-MAP-2 and anti-GFAP, which are specific for neurons and glial cells, respectively. Similar with previous studies (Young and Hung, 2005; Hung and Young, 2006), when FBS was present in the culture medium, most of the differentiated neural stem cells on the control TCPS substrates are immuno-positive for GFAP and immuno-negative for MAP-2 (Figure 4C), indicating that under this condition, neural stem cells are preferably induced to differentiate into glial cells. However, under the same culture condition, neuronal differentiation of neural stem cells can be achieved and increased on the PVA-C substrates at all indicated collagen concentrations (Figure 4C). Quantitatively, the ratio of differentiated MAP-2-immuno-positive neurons on the PVA-C substrates [PVA-C (25 μg/mL), PVA-C (50 μg/mL), and PVA-C (100 μg/mL)] are all significantly higher than that on the control TCPS wells (Figure 4D). Moreover, it is found that the ratio and number of differentiated MAP-2-immuno-positive neurons increase with the decreasing concentration of collagen blended in the PVA-C substrates (Figure 4D). Western blot analysis revealed that the MAP-2 protein expression on PVA-C (25 μg/mL) and PVA-C (50 μg/mL) substrates are higher than the control TCPS group, but is dramatically decreased on the PVA-C (100 μg/mL) substrate (Figure 4E). These results indicate that neuronal differentiation of rat cerebral cortical neural stem cells on the PVA-C substrates can be controlled in a collagen-concentration dependent manner.

### Enriched UCP-1-positive brown adipocyte differentiation of human embryonic stem cells on the PVA-C and PVA-CG substrates

**FIGURE 5:**
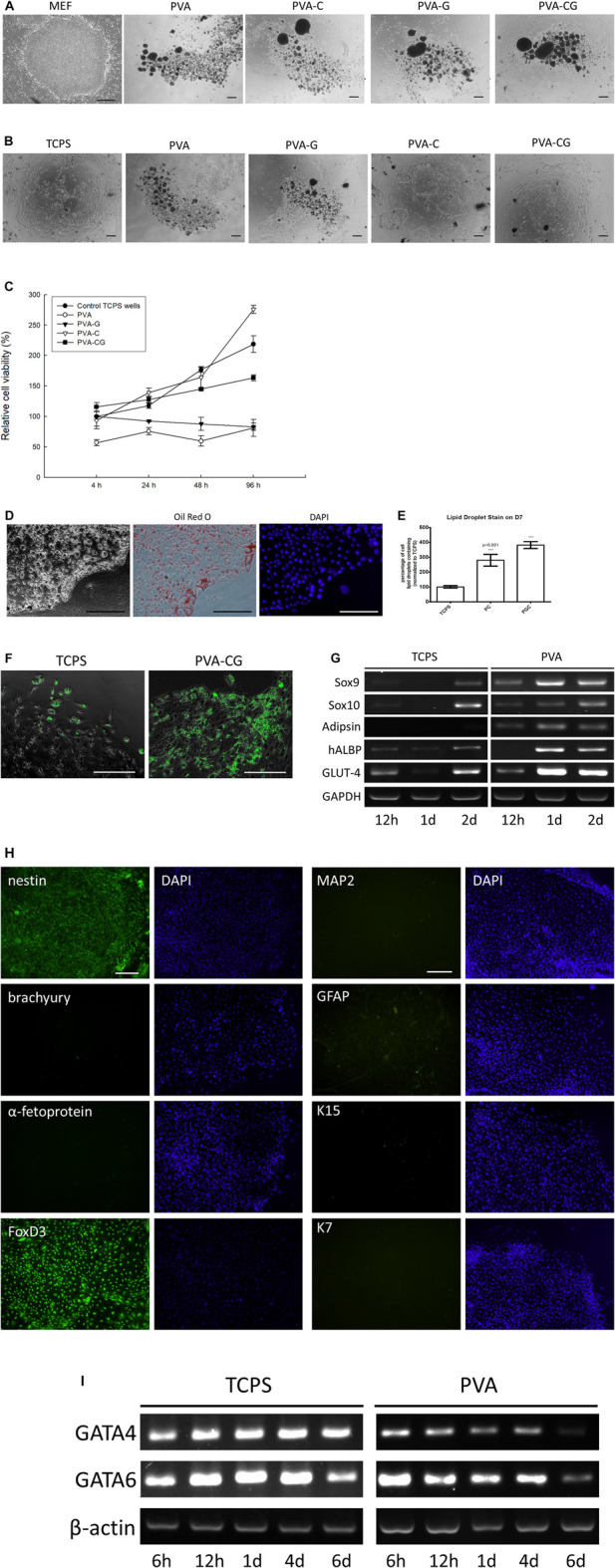
(Continued) Enriched UCP-1-positive brown adipocyte differentiation of human embryonic stem cells on the PVA-C and PVA-CG substrates. **(A)** Clumps of human ES cells cultured on the mouse embryonic fibroblast feeders, PVA, PVA-G, PVA-C, and PVA-CG substrates in the human ES culture medium after 6 days of culture. **(B)** Clumps of human ES cells cultured on the control TCPS wells, PVA, PVA-G, PVA-C, and PVA-CG substrates in the FBS-containing DMEM/F12 after 6 days of culture. **(C)** Relative cell viability of human ESCs on the control TCPS wells, PVA, PVA-G, PVA-C, and PVA-CG substrates in the FBS-containing DMEM/F12 after 4, 24, 48, and 96 h of culture. Graph represents the mean MTT absorbance (O.D. 570–O.D. 630; normalized to the absorbance of the control TCPS wells at 4 h) ± s.e.m. from 6 independent determinations. **(D)** Oil Red O staining of differentiated human ES cells on the PVA-CG substrates after 9 days of culture. **(E)** Relative amount of Oil Red O lipid droplet containing cells derived from human ES cells on the control TCPS wells, PVA-C (PC) and PVA-CG (PCG) polymer substrates after 9 days of culture. *** denotes significant difference (*p* < 0.001) compared to that on the control TCPS wells as determined by Student’s *t*-test. **(F)** Immunocytochemical stainings of UCP-1-positive cells derived from human ES cells on the control TCPS wells and PVA-CG substrates. **(G)** RT-PCR analysis of Adipsin, hALBP, GLUT-4, Sox9, and Sox10 expression of human ESCs cultured on the control TCPS and PVA substrates at indicated time points. **(H)** Immunocytochemical stainings with nestin, brachyury, α-fetoprotein, FoxD3, MAP-2, GFAP, K15, and K7 of differentiated human ES cells on the PVA-CG substrates in the FBS-containing DMEM/F12. **(I)** RT-PCR analysis of GATA4 and GATA6 expression of human ESCs cultured on the control TCPS and PVA substrates at indicated time points. **(I)** Scale bar = 50 μm.

Because of the ability of self-renewing and high plasticity, the pluripotent stem cells are the most promising cells for future cell-based therapies in the field of regenerative medicine and tissue engineering. Here, we also examine the effect of collagen- or/and gelatin-blended PVA substrates on the *in vitro* behavior of pluripotent human ES cells. First, we investigate if these modified PVA substrates can serve as mouse embryonic fibroblast feeder layers to maintain human ES cells in the state of self-renewing. Traditionally, human ES cells can attach and propagate in number without undergoing differentiation on the mouse embryonic fibroblast feeder layers in the ES culture medium; DMEM/F12 containing 20% KnockOut serum replacement (Figure 5A). However, under the same culture condition, it is observed that human ES cells cannot attach but suspend on the PVA, PVA-C, PVA-G, and PVA-CG substrates (Figure 5A), suggested that these non-blended and blended PVA substrates fail to function as mouse embryonic fibroblast feeder layers to anchor and support self-renewing of human ES cells.

Because FBS is an important factor for cells to anchor onto the substrates, we replace the ES culture medium with the FBS-containing medium (DMEM/F12 containing 10% FBS) to see whether human ES cells can attach, grow, and be induced to differentiation on the substrates prepared in this study. It is observed that, after 6 days of culture *in vitro*, the clumps of human ES cells now can attach and grow on the PVA-C and PVA-CG substrates as well as on the control TCPS substrates (Figure 5B). However, even in the presence of FBS in the culture medium, human ES cells cannot attach but suspend on the PVA and PVA-G substrates. According to the results of MTT assay, the viability of human ES cells on the control TCPS, PVA-C, and PVA-CG substrates increased with time and are higher than those on the PVA and PVA-G substrates at indicated time points (Figure 5C)

Morphologically, it is found that human ES cells cultured on the PVA-CG substrates are induced to differentiate into homogeneous populations with multiple Oil-Red-O-positive lipid droplets in the cytoplasm similar to brown adipocytes in appearance (Figure 5D). Quantitatively, the relative amount of differentiated lipid-droplet-containing cells on the PVA-C and PVA-CG substrates are significantly higher than that on the control TCPS wells (Figure 5E). The results of immunocytochemical stainings also demonstrated that most of the differentiated lipid-droplet-containing cells on the PVA-CG substrates are immunoreactive with anti-uncoupling protein 1 (anti-UCP1; a marker specific for brown adipocytes) (Klaus et al., 1991; Ricquier, 2017) and the amount of UCP1-positive cells on the PVA-CG substrates is more abundant than on the control TCPS wells (Figure 5F). The results of RT-PCR also found that as comparing with the control TCPS wells, the PVA substrates increased the expression level of adipocyte-related adipsin, hALBP and GLUT-4 mRNAs of differentiated human ES cells (Figure 5G), which suggested that PVA substrates might favor adipogenic differentiation of human ES cells.

In order to elucidate the lineage specification of human ES cells on the PVA-CG substrates, differentiated cells are immunocytochemically stained with anti-nestin (a ectodermal marker), anti-Brachyury (a mesodermal marker), and anti-α-fetoprotein (a endodermal marker). The results indicated that these differentiated human ES cells on the PVA-CG substrates were immune-positive with anti-nestin, but immune-negative with anti-Brachyury and anti-α-fetoprotein (Figure 5H), which suggested that PVA-CG substrates preferably induce human ES cells differentiation toward ectodermal lineage. The results of RT-PCR analysis also demonstrate that as comparing with control TCPS wells, PVA substrates can downregulate the gene expression of GATA4 and GATA6, an endodermal and mesodermal marker, respectively (Figure 5I). These results assumed that PVA-CG substrates might support ectodermal differentiation but suppress mesodermal and endodermal differentiation of human ES cells. Although adipocytes are thought to be derived from mesoderm generally, Billon et al. (2007) have demonstrated that brown adipocytes could also be derived from neuroectodermal neural crest during embryonic development. Here, it is found that mRNA expression of SOX9 and SOX10, two markers highly expressed in the neuroectodermal neural crest (Cheung and Briscoe, 2003; Haldin and LaBonne, 2010), were enhanced and increased on the PVA substrates with time during 2 days of human ES cell culture (Figure 5G). It is also observed that these differentiated human ES cells on the PVA-CG substrates were immune-positive with FoxD3, an important transcription factor expressed in the ectoderm-derived neural crest (Barembaum and Bronner-Fraser, 2005; Teng et al., 2008), but were immuno-negative with other ectodermal neural (MAP-2), glial (GFAP) and epithelial (K15 and K7) markers (Figure 5H). Taken together, these data suggested that PVA-C and PVA-CG substrates are biocompatible and can serve as suitable substrates to adhere, grow and enrich UCP-1-positive brown adipocyte differentiation of human ES cells through ectodermal lineage.

## Discussion

**FIGURE 6:**
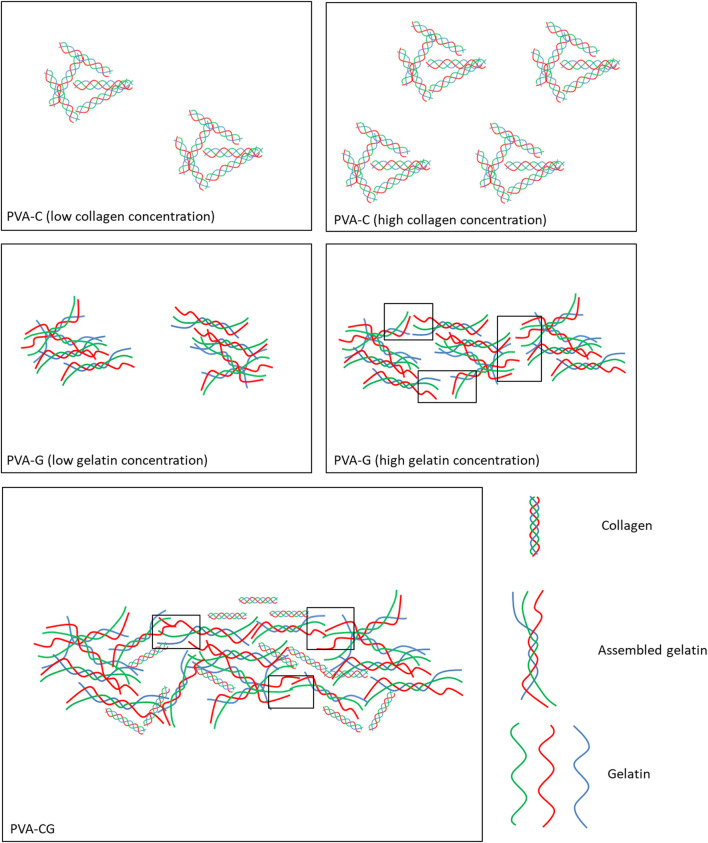
Schemes illustrate the presumption of morphological domain of nucleated particles and elongated fibrils found in the PVA-C, PVA-G, and PVA-CG substrates. The blank rectangles in the figure present the area of new triple helix domain appeared later from assembling of two endpoints of neighboring non-helix gelatin.

Biomaterials play an important role in directing cell behaviors *in vitro* and *in vivo*. In this study, we development a novel culture substrate, which is made from polyvinyl alcohol (PVA) blended with collagen or/and gelatin, to control cell attachment, proliferation, shape, spreading as well as differentiation of stem cells. It is the first time to find that collagen or/and gelatin can self-assemble to form nucleated particles and branched fibrils in the PVA substrates with the scale that can be observed under the optic microscope. It is still unknown if PVA also involved in the assembly of the particles and fibrils. However, we speculate that the PVA polymer solution provide a suitable environment to drive assembly of collagen and gelatin. Because the solvent of PVA, collagen and gelatin in this study are water, it makes collagen and gelatin can freely diffuse and collide to assemble in the PVA polymer solution. Here, we assumed that the nucleated particles in the PVC-C substrates are aggregates of assembled collagen triple helix domain as depicted in Figure 6. The number of aggregates in the PVA-C substrates increases with the collagen concentration. In the PVA-G substrates, the nucleated particles observed in lower gelatin concentration are aggregate of assembled gelatin triple helix domain and the branched and elongated fibrils observed in higher gelatin concentration are assembled from two endpoints of neighboring non-helix gelatin to form new triple helix domain. In the PVA-CG, the fibrils are developed and reinforced from both gelatin and collagen triple helix domain. The PVA-C, PVA-G, and PVA-CG substrates used in this study are all prepared by the method of drying process, a very simple method that can be done in every laboratory. Several parameters, including temperature, periods of drying and intrinsic properties of PVA polymer, will influence the particle and fibril assembly in the PVA polymer substrate, the accessibility for cell culture and the cell behavior *in vitro*. For example, both fully hydrolyzed PVA (BF-17, viscosity: 25–30 cps, hydrolysis: 98.5–99.2 mol %) and partially hydrolyzed PVA (BP-17, viscosity: 21–26 cps, hydrolysis: 86–89 mol %) can be used to prepare PVA-C, PVA-G, and PVA-CG polymer substrates. After blending collagen/gelatin, these fully and partially hydrolyzed PVA showed similar surface structure. However, substrates prepared from fully hydrolyzed PVA will easily swell and distort in the culture medium owing to its higher swelling property. Only substrates prepared from the partially hydrolyzed PVA can be used to culture cells.

Previous studies have shown that, owing to the intrinsic properties of the PVA polymer substrate, several anchoring-dependent cells cannot attach and survive upon this substrate *in vitro* (Nuttelman et al., 2001; Schmedlen et al., 2002; Baker et al., 2012). In this study, it is shown that the attachment of cells (NIH-3T3) can be improved on the modified PVA-C, PVA-G, and PVA-CG substrates. Interestingly, these blended PVA substrates have better cell attachment than traditionally common used TCPS, collagen-coated, and gelatin-coated wells. However, the proliferation of cells on these modified PVA substrates is not comparable with those on the TCPS, collagen-coated, and gelatin-coated wells. We speculated that the reduction of cell proliferation on the PVA-C, PVA-G, and PVA-CG substrates may be caused by the higher adhesion property of the modified substrates. Although we cannot find any direct evidences support the speculation that cell grown on highly adhesive biomaterials exhibit lower cell proliferation. Previous studies have proposed the so-called “receptor saturation” model that cell spreading and motility are suppressed at high collagen density on the substrates since the available integrin receptors on the cell surface are bound to surface abundant collagen ligand focally and become saturated by the ligand on the substrates over a small distance (Schor, 1980; Gaudet et al., 2003). One study also indicated that cells show maximal migration on fibronectin and type IV collagen occurs at an intermediate attachment strength (DiMilla et al., 1993). Similar with the results shown in our study, when cells are cultured on the PVA substrates blended with higher amount of collagen, their spreading and motility are restricted, which lead to the cells exhibiting oval morphology and simultaneously inhibit their proliferation. One recent study had also proved that fibrillar collagen but not the monomer collagen inhibits arterial smooth muscle proliferation through regulation of Cdk2 inhibitors (Koyama et al., 1996), which similar with our results in this study that inhibited cell proliferation are observed on collagen or/and gelatin assembling macroscale structure on the PVA-C, PVA-G, and PVA-CG substrates which exhibits higher cell adhesion property at the same time. It is also noticed that although Figures 2A, B demonstrated that adhesion of NIH-3T3 cells to PVA-C was better than to PVA-G, but Figures 2D, E showed that the values of MTT test on PVA-C (∼200%) was greater than PVA-G (<150%) after 4 days of culture. The possible reasons of these conflicts may be caused by: 1. the initial cell adhesion at 4 h on PVA-C substrates is 2 to 3 fold higher than that on PVA-G substrates; 2. the cells may continuously attach after 4 h of culture and proliferate a little bit after 1 day, 2 days, and 4 days of culture on the PVA-C substrates; 3. the value of MTT test after 1 day, 2 days, and 4 days of culture cannot use as an estimate of viable cell number but the viability and growth of cells, since these cells exhibited different types of morphology on the PVA-C and PVA-G substrates. We assumed that the higher value of MTT test on PVA-C (∼200%) after 4 days of culture is contributed from the higher cell adhesion in the beginning but not from the cell proliferation. Since cell proliferation involves adhesion, spreading and migration of cells, we reasonably suspected that the higher collagen concentration which caused more fibril formation as well as more receptor saturation may result in the increased cell adhesion and inhibited cell spreading and proliferation. Besides biological factors originated from the nature of collagen and gelatin, physical factors such as surface topography, mechanical properties, electric field, surface charges, water content and so on can influence cell adhesion, spreading and proliferation. Results in this study also suggested that formation of particles and fibrils in the PVA-C, PVA-G, and PVA-CG substrates increases the substrates’ roughness and Young’s modulus as well as the adhesion and viability of cells as comparing with pure PVA substrates. However, further studies are needed to confirm whether biochemical factors (originated from the nature of collagen/gelatin and the particle/fibril formation) or physical factors (originated from and the topographical and mechanical difference) has a greater influence on cell adhesion, spreading and proliferation.

Another interesting finding in this study is that the shape of NIH-3T3 cells cultured on the blended PVA-CG substrates can be switched between oval, spindle and flatten spreading morphologies depending on the amount of collagen and gelatin in the PVA substrates. Generally, cells tend to exhibit oval or flatten spreading morphology when the PVA-CG substrates are blended with higher amount of collagen or gelatin, respectively. Otherwise, intermediate spindle morphology of cells can be obtained when the PVA-CG substrates are blended with lower amount of collagen or gelatin. Here, we reasonably assumed that the presence of collagen may inhibit cell spreading and induce the cells to exhibit the oval morphology; on the contrary, the presence of gelatin may counteract the effect of collagen and drive the cells to spread into the flatten morphology. It is well known that changes in cell shape play an important role for tissue morphogenesis during development, and the alternation between these morphological appearances might also change their intrinsic functions such as cell migration, proliferation, differentiation, gene expression or mesenchymal-to-epithelial transition of cancer cells, etc (Meyers et al., 2006; Paluch and Heisenberg, 2009; Prasad and Alizadeh, 2019). Hence, the results of this study may provide a clue to design an appropriate culture substrate to control cell shapes as well as their functions *in vitro*.

Developing an appropriate niche, either prepared from naturally-derived or synthetic biomaterials, for conducting or supporting differentiation of stem cells toward specific cell types remains a fascinating and obscure issue in the field of regenerative medicine. In this study, we have examined the influence of the modified PVA-C, PVA-G, and PVA-CG substrates on the differentiation of rat cerebral cortical neural stem cells and human ES cells. It is found that without the presence of FBS in the culture medium, rat neural stem cells cannot attach and differentiate on all of these modified PVA substrates. However, when FBS is present in the medium, rat neural stem cells can only attach and differentiate on the PVA-C substrates but not on the PVA-G and PVA-CG substrates. These results suggested that FBS is essential for cell attachment and the gelatin blended in the modified PVA substrates may have inhibitory effect on attachment of rat neural stem cells. Moreover, the morphological appearance as well as their differentiation of rat neural stem cells on the PVA-C substrates is depending on the amount of collagen blended in the PVA substrates. When the collagen blended in the substrates is high, these neurospheres stay intact and reveal round and spheroid morphology analogous to the oval morphology of NIH-3T3 observed in this study. At this time, the neural stem cells within the neurospheres cannot spread and migrate out to differentiate. However, after decreasing the amount of collagen blended in the PVA substrates, the neurospheres start to spread and a great amount of cells migrate out from the neurospheres and exert a more spindle and stretching morphology. It is well known that MAP-2 protein, is thought to be involved in cytoskeleton microtubule assembly, plays an important role in neurite growth and neurogenesis (Harada et al., 2002; Huang et al., 2013). The Western blot analysis in Figure 4E confirmed that enhanced MAP-2 protein expression on PVA-C (25 μg/mL) and PVA-C (50 μg/mL) substrates were observed since the neurons differentiated on these substrates adopt a more extending morphology and develop more neurite growth as shown in Figure 4C. On the other hand, low MAP-2 protein expression on PVA-C (100 ug/mL) indicated reduced spreading and neurite grwoth of differentiating cells.

Owing to their potential of providing unlimited number of cells and differentiating into almost all cell types, embryonic stem cells are the most promising cell source for therapeutic application and tissue regeneration. Up to date, several methods including embryoid-body formation, cultures on feeder cells and cultures on naturally-derived or synthetic substrates, have been utilized to induce or support human ES cell differentiation to the committed cells *in vitro* (Bratt-Leal et al., 2009; Lutolf et al., 2009). Current protocols of human ES cell differentiation into brown adipocytes fall into two categories (Carobbio et al., 2021). The first relies on ectopic overexpression of genes that drive the brown adipocyte program. For example, Ahfeldt et al. (2012) demonstrated overexpression of PPARG2 combined with CEBPB and PRDM16 in mesenchymal progenitor cells derived from human pluripotent stem cells programmed their development towards brown adipocyte cell fate with efficiencies of 85%–90%. The second relies on derivation of mesenchymal progenitor cells or embryoid bodies before applying a chemical adipogenic stimulus. For example, Nishio et al. (2012) established a method for a high-efficiency (>90%) differentiation of human pluripotent stem cells into functional classical brown adipocytes using specific hemopoietin cocktail. In this study, hemopoietin cocktail I was used to efficiently direct human ES cells differentiation towards mesodermal mesenchymal progenitor cells in 8 days and hemopoietin cocktail II was used to induce brown adipocytes from human ES cell-derived mesenchymal progenitor cells in additional 7 days. Several other studies have also revealed an important role of BMP7 in promoting brown adipocyte differentiation of mesodermal mesenchymal progenitor cells *in vitro* and *in vivo* (Tseng et al., 2008; Shaw et al., 2021). However, these differentiated brown adipocytes are thought to be derived from mesodermal progenitors. Little is known which factors are important for directing human ES cells differentiation into brown adipocytes via ectodermal progenitor and whether BMP7 is also involved in this process is still unkown. Generally, adipocytes are believed to be mesodermal in origin, but it is worth noting that in higher vertebrate, the mesoderm is not the only germ layer source of mesenchymal cells. In the development of the neural crest, which is the ectodermal-derived mesenchymal cells, yields mesectodermal cells that can differentiate into connective tissue cells, vascular smooth muscle cells, cartilage, bone, as well as adipocytes (Bronner and LeDouarin, 2012; Weston and Thiery, 2015). Billon et al. (2007) have demonstrates that neuroectoderm/neural crest may be a source of adipocytes in mouse ES cells-derived culture and in quail embryos, although the authors did not distinguish these differentiated adipocytes are white or brown adipocytes in that study. For now, little is known whether brown adipocytes can also be derived from ectodermal progenitors. In this study, it is the first time to find that human ES cells can be induced into brown adipocytes through the ectodermal lineage. Since the results in Figures 5G–I suggested that PVA substrates promote ectodermal development as well as inhibit mesodermal and endodermal development of human ES cells, we speculate that the differentiated UCP1-immunopositive brown adipocytes by our differentiation method are derived from ectodermal linage, rather than from mesodermal linages. It is necessary to state that the biochemical factors that trigger human ES cells to yield brown adipocytes via ectodermal lineage in this study remain unclear. However, the physical factor stiffness of PVA substrates may be one factor that affects the brown adipocyte differentiation of human ES cells since the soft PVA hydrogel may show comparable stiffness as native adipose tissue. Moreover, the methods published previously to direct brown adipocyte differentiation from pluripotent stem cells or multipotent adipose-derived stem cells need various inducing factors and require long-term culture (Elabd et al., 2009; Hafner and Dani, 2014; Mohsen-Kanson et al., 2014). Here, we provide a relative simple and inductive factor-free method to enrich brown adipocyte differentiation from human pluripotent ES cells in 9-day culture. These results provide a valuable culture platform to elucidate the molecular basis of adipocyte development of human ES cells.

## Data Availability

The original contributions presented in the study are included in the article/supplementary material, further inquiries can be directed to the corresponding author.
